# Comparison of membrane proteins of *Mycobacterium tuberculosis *H37Rv and H37Ra strains

**DOI:** 10.1186/1471-2180-11-18

**Published:** 2011-01-24

**Authors:** Hiwa Målen, Gustavo A De Souza, Sharad Pathak, Tina Søfteland, Harald G Wiker

**Affiliations:** 1 Section for Microbiology and Immunology, the Gade Institute, University of Bergen, Bergen, Norway; 2 Department of Microbiology and Immunology, Haukeland University Hospital, Bergen, Norway; 3 Proteomic Unit, Department of Biomedicine, University of Bergen, Norway

## Abstract

**Background:**

The potential causes for variation in virulence between distinct *M. tuberculosis *strains are still not fully known. However, differences in protein expression are probably an important factor. In this study we used a label-free quantitative proteomic approach to estimate differences in protein abundance between two closely related *M. tuberculosis *strains; the virulent H37Rv strain and its attenuated counterpart H37Ra.

**Results:**

We were able to identify more than 1700 proteins from both strains. As expected, the majority of the identified proteins had similar relative abundance in the two strains. However, 29 membrane-associated proteins were observed with a 5 or more fold difference in their relative abundance in one strain compared to the other. Of note, 19 membrane- and lipo-proteins had higher abundance in H37Rv, while another 10 proteins had a higher abundance in H37Ra. Interestingly, the possible protein-export membrane protein *SecF *(Rv2586c), and three ABC-transporter proteins (Rv0933, Rv1273c and Rv1819c) were among the more abundant proteins in *M. tuberculosis *H37Rv.

**Conclusion:**

Our data suggests that the bacterial secretion system and the transmembrane transport system may be important determinants of the ability of distinct *M. tuberculosis *strains to cause disease.

## Background

Tuberculosis is an airborne infection caused by *M. tuberculosis*. It is estimated that one-third of the world's population is latently infected with *M. tuberculosis*, and that each year about three million people die of this disease. The emergence of drug-resistant strains is further worsening the threat (WHO, 2003). In spite of global research efforts, mechanisms underlying pathogenesis, virulence and persistence of *M. tuberculosis *infection remain poorly understood [1].

A central issue in the pathogenesis of tuberculosis is the characterization of virulence determinants of *M. tuberculosis *that are relevant to human disease [2]. Attenuated strains of mycobacteria can be exploited to determine genes essential for pathogenesis and persistence. The best studied virulent laboratory strain of *M. tuberculosis *H37Rv has an avirulent counterpart in *M. tuberculosis *H37Ra, which was recognized as early as 1934 [3]. Though infectious, it does not replicate in macrophages [4] and resembles the dormancy of *M. tuberculosis *during latent infection. Reasons for the decreased virulence remain incompletely understood [5]. The genetic and phenotypic differences between these strains have been subject to intensive investigation in an attempt to identify virulence determinants. As a result, some genes have been found; for example, the *eis *(enhanced intracellular survival) gene and *erp *(exported repetitive protein) genes enhance *M. tuberculosis *survival in macrophages [6,7], *ivg *(in vivo growth) of *M. tuberculosis *H37Rv confers a more rapid in vivo growth rate to *M. tuberculosis *H37Ra [8]. Aside from the identified virulence factors, genomic differences such as insertions, deletions and single nucleotide polymorphisms have been found in both virulent and attenuated *Mycobacteria *[9]. Irrespective of genomic differences between H37Ra and H37Rv, other studies investigated the phenotypic consequences and determined changes in gene expression. Gao et. al. (2004) performed a genome-wide approach using microarrays to compare the transcriptomes of *M. tuberculosis *H37Rv and *M. tuberculosis *H37Ra [10]. Many genes whose expression was repressed in *M. tuberculosis *H37Ra were discovered. Hence, although it is important to identify genes related to *M. tuberculosis *virulence, attention should also be paid to the gene products at protein level being responsible for virulence. Proteomics characterization represent an important complement to genomics in showing which genes are really expressed. Improved label-free approaches have recently provided a new dimension to proteomic methods [11]. The proteome of BCG can reveal proteins that are differentially expressed including up-regulation and down-regulation under standing and shaking culture conditions [12]. This can not be elucidated using genomic analysis. Additionally, proteomics of *M. tuberculosis *H37Rv has revealed six open reading frames not predicted by genomics [13]. Differences in protein composition between attenuated strains and virulent *M. tuberculosis *are helpful for the design of novel vaccines and chemotherapy.

*M. tuberculosis *is a facultative intracellular pathogen that resides within the host's macrophages [14-16]. When *M. tuberculosis *invades host cells, the interface between the host and the pathogen includes membrane- and surface proteins likely to be involved in intracellular multiplication and the bacterial response to host microbicidal processes [16]. Recently, the cell wall of *M. tuberculosis *was reported to posses a true outer membrane adding more complexity with regard to bacterial-host interactions and also important information relevant for susceptibility to anti-mycobacterial therapies [17-19].

In the present study, we used orbitrap mass spectrometry technology in combination with relative protein expression abundance calculations to compare the membrane protein expression profiles of *M. tuberculosis *H37Rv and its attenuated counterpart H37Ra. The aim was to find proteins that may further explain the different phenotypes of the two strains, especially their distinct ability to cause disease.

## Methods

### Bacterial strains

The two mycobacterial reference strains, *M. tuberculosis *H37Ra (MNC 16394) and *M. tuberculosis *H37Rv (ATCC 27294), used in this study were kindly provided by Dr Harleen Grewal, The Gade Institute, University of Bergen, Norway. The strains had undergone less than 3 passages in the laboratory before being used for this study. The bacilli were cultured on Middelbrook 7H10 agar plates with OADC enrichment (BD Difco) at 37°C and 5% CO_2 _for 3-4 weeks. Bacterial colonies were harvested by using an extraction buffer consisting of phosphate-buffered saline (PBS), pH 7.4 with freshly added Roche Protease Inhibitor Cocktail (1 μg/ml) (Complete, EDTA-free, Roche Gmbh, Germany). Six hundred μl of this extraction buffer was added to each agar plate and the mycobacterial colonies were gently scraped off the agar surface using a cell scraper. Aliquots of the resulting pasty bacterial mass were transferred into 2 ml cryotubes with O-rings (Sarstedt, Norway) containing 250 μl of acid washed glass beads (≤106 μm; Sigma-Aldrich, Norway) and an additional 600 μl of extraction buffer containing a cocktail of protease inhibitors (1 μg/ml) (Roche Diagnostics GmbH), and stored at -80°C until further treatment. For protein extraction, the mycobacteria were disrupted mechanically by bead-beating in a Ribolyser (Hybaid, UK) at max speed (6.5) for 45 seconds.

### Triton X-114 extraction of exported proteins from whole bacteria

Triton X-114 phase-partitioning was used to isolate lipophilic proteins following the method of Bordier [20] and a modified version for extraction of lipophilic proteins from whole bacilli [21]. Briefly, 3-4 week old bacilli were lysed by bead-beating and unbroken cells and cell-wall debris were removed by centrifugation at 2300 *g *for 5 minutes. Triton X-114 was added to the supernatant (final detergent concentration 2%, w/v) and the suspension was stirred at 4°C for 30 minutes. Residual insoluble materials were removed by centrifugation at 15700 *g *for 10 min at 4°C. For separation of the hydrophobic and hydrophilic proteins, the solution was incubated at 37°C for 15 minutes, the solution separated into two phases, an upper aqueous phase containing hydrophilic proteins, and a lower (detergent) phase containing the hydrophobic proteins. Proteins in the lower detergent phase were precipitated by acetone.

### Gel electrophoresis and in-gel digestion of proteins

Extracted proteins, 50 μg from each strain, were mixed with 25 μl sodium-dodecyl-sulphate (SDS) loading buffer and boiled for 5 minutes before separation on a 10 cm long 1 mm thick 12% SDS polyacrylamide gel. The protein migration was allowed to proceed until the bromophenol dye had migrated to the bottom of the gel. The protein bands were visualized with Coomassie Brilliant Blue R-250 staining (Invitrogen, Carlsbad, CA, U.S.A.). Protein lanes were divided in 10 fractions by cutting between the visible protein bands ranging from ~3 kDa to ~188 kDa and washed twice for 15 minutes at room temperature (RT) with 50% acetonitrile (ACN) in 25 mM ammonium bicarbonate (NH_4_HCO_3_). The gel pieces were dehydrated by incubating them with 50 μl 100% ACN for 20 minutes at RT. The disulfide bonds in the proteins were reduced using 10 mM dithiotreitol and alkylated with 55 mM iodoacetamide; both in 100 mM NH_4_HCO_3_. The gel pieces were dehydrated by 100% ACN as described above, and rehydrated in 25 mM NH_4_HCO_3_. The proteins were digested by trypsin (Promega, Madison, U.S.A.) for 16-20 h at 37°C. The peptides were eluted stepwise from each gel piece using 1% formic acid (FA), then 0.1% FA in 50% ACN and the last one 100% ACN. Each incubation was performed for 20 minutes at RT in 100 μl volumes, and finally the 3 supernatants were pooled.

### Mass spectrometry

Experiments were performed on a Dionex Ultimate 3000 nano-LC system (Sunnyvale CA, USA) connected to a linear quadrupole ion trap-Orbitrap (LTQ-Orbitrap) mass spectrometer (ThermoElectron, Bremen, Germany) equipped with a nanoelectrospray ion source. The mass spectrometer was operated in the data-dependent mode to automatically switch between Orbitrap-MS and LTQ-MS/MS acquisition. Survey full scan MS spectra (from m/z 400 to 2,000) were acquired in the Orbitrap with resolution R = 60,000 at m/z 400 (after accumulation to a target of 1,000,000 charges in the LTQ). The method used allowed sequential isolation of the most intense ions (up to five, depending on signal intensity) for fragmentation on the linear ion trap using collisionally induced dissociation at a target value of 100,000 charges.

For accurate mass measurements the lock mass option was enabled in MS mode and the polydimethyilcyclosiloxane (PCM) ions generated in the electrospray process from ambient air (protonated (Si(CH_3_)_2_O)6; m/z 445.120025) were used for internal recalibration during the analysis [22]. Target ions already selected for MS/MS were dynamically excluded for 30 seconds. General mass spectrometry conditions were: electrospray voltage, 1.9 kV. Ion selection threshold was 500 counts for MS/MS, an activation Q-value of 0.25 and activation time of 30 milliseconds was also applied for MS/MS.

All acquired data were processed and analyzed using MaxQuant (version 1.0.13.13), a software script specifically developed for data acquired using high-resolution instrumentation [23]. MS/MS peak lists from 60 individual RAW files were generated using the Quant.exe tool from the MaxQuant package. Protein identification was performed by searching combined data from each fraction against an in-house developed *M. tuberculosis *complex database (4,643 protein sequences) [24]. The database was also modified to contain reversed sequences of all entries as a control of false-positive identifications during analysis [25]. In addition, common contaminants such as keratins, bovine serum albumin and trypsin were also added to the database (database final size of 9,308 protein sequences). We used MASCOT Deamon for submission of multiple searches to a local Mascot server v2.2 (Matrix Science). The search parameters were: Enzyme: trypsin with no proline restriction; Maximum missed cleavages: 3; Carbamidomethyl (C) as fixed modification; N-acetyl (Protein), oxidation (M), Pyr-Q (Gln to 2-pyrrolidone*-5-*carboxylic acid-Glu) and Pyr-E (Glu to 2-pyrrolidone*-5-*carboxylic acid-Glu) as variable modifications; Peptide mass tolerance of ± 15 parts per million; MS/MS mass tolerance of 0.5 Da.

Protein identification and validation was performed with Identify.exe from MaxQuant using the following parameters: peptide and protein false discovery rate: 0.01 (1%), minimal peptide length was 7, and to guarantee a high confidence identification rate, the maximal posterior error probability was set to 0.1 (from a range of 0 to 1); minimal number of unique peptides per protein: 1. The average mass accuracy for the identified peptides was 400 parts per billion. The MS/MS fragments assignments for all identified peptide sequences (including for single peptide-based protein identifications) are freely available at the Tranche network http://proteomecommons.org (see Supporting Information Available section for more details).

### Estimation of protein abundance

To determine differentially represented membrane proteins between the *M. tuberculosis *H37Rv and the *M. tuberculosis *H37Ra strains, we used MaxQuant peak intensity calculations as a parameter for protein abundance. Previous reports demonstrate a good correlation between peak intensity and protein levels in the sample [26,27]. To avoid variation due to loading differences between samples on the instrument, individual intensity values of each protein were divided by the sum of all intensities in the sample as a normalization procedure.

Proteins were divided in two categories as follows: I) for proteins identified in both samples, the difference in relative abundance between the strains had to be higher than 5 fold; II) for a protein identified in only one of the strains, we required that it had to be identified with a minimal of four different peptides. Such stringent criteria are required to guarantee that a protein identified in only one sample is most probably due to differences in abundance between the samples, and not because parent ions were not identified (but still present) in the MS analysis due to random fluctuation of the MS/MS data-dependant acquisition procedure.

### Primary sequence analysis

The primary sequence analysis of the observed proteins to identify exported proteins were performed using the publically available algorithms: TMHMM version 2 for identification of transmembrane helixes (TMH) in membrane proteins http://www.cbs.dtu.dk/services/TMHMM/, SignalP for prediction of secreted proteins http://www.cbs.dtu.dk/services/SignalP/, and PROSITE for prediction of lipoproteins http://au.expasy.org/prosite/.

## Results

### Triton X-114 detergent extracted proteins

The aim of this study was to perform a proteomic analysis on protein expression of two closely related lineages of *M. tuberculosis*, the virulent H37Rv and the avirulent H37Ra strains, with a main focus on membrane- and membrane-associated proteins. For this purpose, cultured bacilli were mechanically disrupted and proteins extracted by Triton X-114 detergent phase separation. Proteins were then precipitated by acetone, separated by SDS-PAGE, and analysed by high resolution mass spectrometry. Additional Figure 1 gives an example of the quality of the mass spectrometry data gathered in this work, which illustrates the full sequence obtained for ion m/z 1476.82, which was identified by Mascot as peptide LVLGSADGAVYTLAK from Rv2138, probable conserved lipoprotein LppL, with a Mascot score of 118 and contains fragmentation data for all the expected y-series daughter ions. In total, 1771 different protein groups were identified, with 1578 proteins identified in the *M. tuberculosis *H37Rv strain, and 1493 were observed in the H37Ra strain. The additional files 1 &2 include peak lists, information about the criteria of protein identifications, such as number of peptides matching each protein, score and identification threshold.

**Figure 1:**
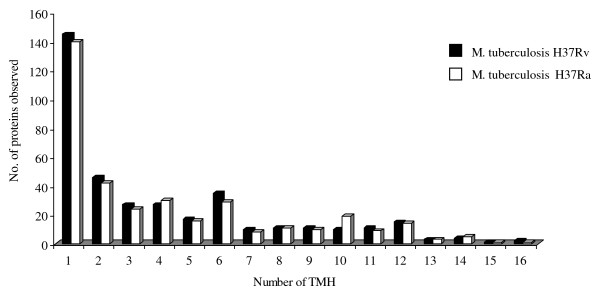
**Identified membrane protein distributions in *M. tuberculosis *H37Rv and H37Ra strains**.

Among the 1771 proteins observed in this study, there were 1300 proteins that were common to both strains. However, 278 proteins were exclusively identified in the *M. tuberculosis *H37Rv, while another 193 proteins were solely observed in the H37Ra strain. Further, to ascertain the validity of the comparison analysis of the two strains due to technical error margins, we have only taken into account the proteins observed with 4 or more different peptides. Using these stringent criteria, we reduced the number of the observed strain specific proteins drastically to only 4 identified in *M. tuberculosis *H37Rv but not observed in H37Ra. Two of them were predicted with 3 (Rv3479) and 13 transmembrane regions (Rv3792), one hypothetical protein (Rv2319c) and one secreted protein (R1184c). No such examples were found in *M. tuberculosis *H37Ra.

The data obtained in this study, was searched for membrane and membrane-associated proteins by using the TMHMM v2.0 algorithm http://www.cbs.dtu.dk/services/TMHMM/. In *M. tuberculosis *H37Rv 371 proteins were identified that were predicted to have 1 or more TMH regions, while in *M. tuberculosis *H37Ra 357 proteins were identified predicted to be anchored to the membrane by 1 or more TMHs. As it appears from Figure 1, the distributions of proteins identified with different number TMHs were similar for the two strains, with proteins with only 1 TMH as the largest group. Three hundred and twenty one of all the membrane proteins were common for both strains, while 36 membrane proteins were only observed in *M. tuberculosis *H3Ra and 51 membrane proteins only observed in *M. tuberculosis *H37Rv (Additional file 3).

Another interesting group of proteins that are associated with the membrane is lipoproteins. These are proteins translocated to the cell membrane and retained there by post-translational lipid modification. They are functionally diverse, and are suggested to be involved in host-pathogen interactions [28,29]. They are also interesting with respect to development of serodiagnostic tests for detection of TB due to their strong immunogenicity [30,31]. Lipoproteins represent a subgroup of secreted proteins characterized by the presence of a lipobox. The lipobox motif is located in the distal C-terminal part of the N-terminal signal peptide [32]. This motif functions as a recognition signal for lipid modification, which is made on the conserved and essential cysteine residue. Precursor lipoproteins are mainly translocated in a Sec-dependent manner across the plasma membrane and are subsequently modified [33]. The proteins identified in this study were analysed by PROSITE for prediction of lipoproteins http://au.expasy.org/prosite/. Seventy-six of them were predicted as potential lipoproteins, based on the presence of a cleavable signal peptide and signal peptidase II recognition motif. Sixty six of all the lipoproteins were common for both strains, while 7 lipoproteins were only observed in *M. tuberculosis *H3Ra and 3 lipoproteins only observed in *M. tuberculosis *H37Rv (Additional file 4).

### Estimation of relative abundance

**Figure 2:**
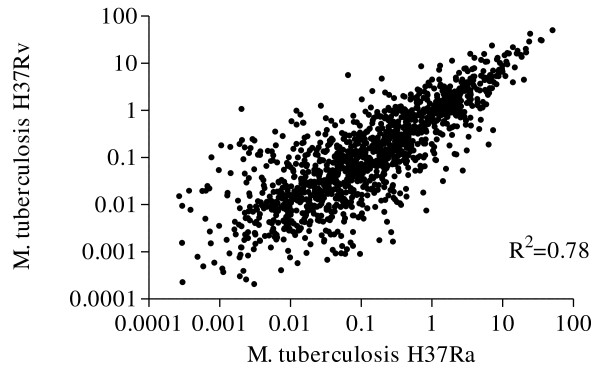
**Illustration of the relative abundance values of each protein observed in both *M. tuberculosis *H37Rv and *M. tuberculosis *H37Ra strains**.

Using MaxQuant software that provide quantitative information about proteins and peptides using the spectra generated during the LC runs the relative abundance of each protein observed in both *M. tuberculosis *H37Rv and *M. tuberculosis *H37Ra were examined after normalization. Our data showed that most of the proteins identified in both strains had similar relative abundance. Using Pearson's method for correlation, the relative abundance of proteins observed in the two strains were significantly correlated with a correlation coefficient of 0.887 (p < 0.001), and R^2 ^= 0.78 (Figure 2). However, there were some proteins that had different relative abundance between the two strains. To ensure the relative protein abundance for these proteins were real and not due to technical error margins, we only focused on the ones with a 5 fold difference or higher. To this end, there were 121 proteins from both strains that belonged to different functional groups (Additional file 5). In order to reduce the amount of data required to be analysed, and due to the anticipated important biological role of membrane- and membrane-associated proteins, we chose to focus only on membrane- and lipoproteins. This further reduced the number of proteins to only 19 and 10 proteins in *M. tuberculosis *H37Rv and *M. tuberculosis *H37Ra, respectively (Table 1). Among the proteins observed with a 5 fold or higher relative abundance in *M. tuberculosis *H37Rv strain, 17 were predicted to be directly anchored to the membrane by TMH retention regions in their primary sequences, while 2 others are thought to be retained on the membrane through N-terminal lipidation.

**Table 1:** List of *M. tuberculosis *H37Rv and *M. tuberculosis *H37Ra proteins, with difference in relative abundance of 5 fold or higher.

*Protein IDs*	*Protein description*	*Gene Name*	*Functional group*	*Ratio H37Rv/H37Ra*	*Ratio H37Ra/H37Rv*	*TM number*	*References*
Rv0319	Probable conserved integral membrane protein	-	3	-	6 ^b^	8 ^c^	
Rv1101c	Conserved membrane protein	-	3	-	5	8	[21,60]
Rv1030	Probable potassium-transporting p-type	-	3	-	12	7	
Rv2560	Probable proline and glycine rich transmembrane	-	3	-	24	4	[21]
Rv2732c	Probable conserved transmembrane protein	-	3	-	7	4	
Rv0014c	Transmembrane serine/threonine-protein kinase b	-	9	-	18	1	[21]
Rv3584	Possible conserved lipoprotein	*lpqe*	3	-	11	1	[21,60-63]
Rv3869	Possible conserved membrane protein	-	3	-	6	1	
Rv0070c	Probable serine hydroxymethyltransferase	*glya2*	7	-	82	0	[64]
Rv3576	Possible conserved lipoprotein	*lpph*	3	-	11	0	[21]
Rv0402c	Probable conserved transmembrane transport	-	3	7^a^	-	12	[61,64]
Rv0933	Phosphate-transport ATP-binding ABC transporter	*pstB*	3	106	-	0	
Rv3273	Probable transmembrane carbonic anhydrase	-	7	33	-	10	[60,62,63]
Rv2051c	Polyprenol-monophosphomannose synthase	*ppm1*	3	22	-	7	[63,64]
Rv2877c	Probable conserved integral membrane protein	-	3	5	-	7	
Rv1273c	Probable drugs-transport transmembrane	-	3	7	-	6	
Rv1819c	Probable drugs-transport transmembrane	-	3	6	-	6	[60,63,64]
Rv2586c	Probable protein-export membrane protein	*secf*	3	7	-	6	[21,60,63]
Rv1779c	Hypothetical integral membrane protein	-	3	21	-	4	[64]
Rv2197c	Probable conserved transmembrane protein	-	3	8	-	4	[21,63]
Rv2617c	Probable transmembrane protein	-	3	11	-	3	
Rv0284	Possible conserved membrane protein	-	3	11	-	1	[60,63,64]
Rv0291	Probable membrane-anchored mycosin	*mycp3*	7	6	-	1	[60-63]
Rv1209	Conserved hypothetical protein	-	10	19	-	1	[21,63]
Rv1885c	Conserved hypothetical protein	-	10	7	-	1	[21]
Rv2289	Probable cdp-diacylglycerol pyrophosphatase	*cdh*	1	42	-	1	[21,60,63]
Rv0265c	Probable periplasmic iron-transport lipoprotein	-	3	7	-	0	[21,61-63]
Rv0583c	Probable conserved lipoprotein lpqn	lpqn	3	19	-	0	[21,60,61,63]
Rv2833c	Probable sn-glycerol-3-phosphate-binding	-	3	9	-	0	[21,64]

^a ^Proteins more abundant in M. tuberculosis H37Rv strain compared to H37Ra strain. Relative abundance ratio calculated based on intensity measurements performed using MSQuant algorithm http://msquant.sourceforge.net/.

^b ^Proteins more abundant in M. tuberculosis H37Ra strain compared to H37Rv strain. Relative abundance ratio calculated based on intensity measurements performed using MSQuant algorithm http://msquant.sourceforge.net/.

^c ^Number of transmembrane regions predicted in the primary amino acid sequence by TMHMM v 2.0 http://www.cbs.dtu.dk/services/TMHMM/.

## Discussion

Due to the anticipated importance of membrane- and membrane-associated proteins of *M. tuberculosis *in bacterial virulence, it is essential to map these proteins. Therefore, the aim of this study was to characterize the repertoire of membrane and membrane associated proteins from the two widely used *M. tuberculosis *strains H37Rv (virulent) and H37Ra (avirulent). As the *M. tuberculosis *H37Ra genome has recently been sequenced, there is currently great interest in investigating the differences between the two strains in more detail [34-36]. The protein profile data of the two strains were further analysed with the aim of finding relative quantitative differences of the observed proteins. Using proteomic data to quantify proteins gives a more realistic impression about the protein content and hence the physiological state of the bacilli, rather than mRNA measurement, as mRNA levels do not necessarily reflect the amount of proteins expressed. High-throughput proteomics using state-of-art instruments is well suited for providing more detailed information of the differences in expressed proteins between the two strains, complementing and adding to prior studies that have mainly focussed on gene expression by mRNA measurements [10,36].

We observed that the vast majority of the proteins were present in both strains and had similar relative abundance (Figure 2). This was expected as the two strains are closely related. However, a small group of proteins had a different relative abundance in the two strains.

Among the differently abundant proteins, a member of the general secretory (Sec) pathway (Rv2586c, *SecF*) was identified with over 6 fold higher relative abundance in *M. tuberculosis *H37Rv compared to *M. tuberculosis *H37Ra (Table 1). In bacteria, the bulk of protein export across the cytoplasmic membrane is carried out by this pathway [37-39]. The final destination of Sec exported proteins can be the cell envelope or the extracellular space. The Sec pathway is well-characterized in *Escherichia coli *[37,38,40]. At the core of the Sec pathway is a membrane-spanning translocation channel composed of the integral membrane proteins: Rv0638 (*SecE1*), Rv0379 (*SecE2*), Rv2586c (*SecF*), Rv1440 (*SecG*), Rv0732 (*SecY*) [41]. *SecA *binds to cytoplasmic precursor proteins destined for export and delivers them to the translocation machinery through its ability to bind to membrane phospholipids [42]. The three subunits with predicted transmembrane regions that comprise the core of the Sec translocation and export machinery are all identified in both strains. The two other components, Rv0732 (*SecY*) and Rv2587c (*SecD*), also have higher relative abundance in *M. tuberculosis *H37Rv. Since we restricted the analysis only to the ones with 5 fold difference or more, these were not included in the Table 1. Nevertheless, our data indicates a trend of higher expression of these subunits.

Three proteins (Rv0933, Rv1273c and Rv1819c) belonging to transmembrane ATP-binding ABC transporter proteins were observed with >5x higher relative abundance in the *M. tuberculosis *H37Rv. ABC transporter proteins are found in both eukaryotes and prokaryotes and constitute a large super family of multi-subunit permeases that transport various molecules (ions, amino acids, peptides, antibiotics, polysaccharides, proteins, etc.) across biological membranes, with a relative specificity for a given substrate [43]. They consist of two hydrophobic membrane spanning domains (MSDs) associated with two cytoplasmic nucleotide binding domains (NBDs) [44-46]. They are classified as importers and exporters depending on the direction of translocation of their substrate [47]. Importers are found exclusively in prokaryotes and are involved in the uptake of extracellular molecules [48]. Exporters are found in both prokaryotes and eukaryotes, where they export molecules from the cytoplasm [49]. Taken together, the observation of three transporter proteins with higher abundance in *M. tuberculosis *H37Rv may suggest a significant role of these proteins in the overall transport of nutrition by the bacilli, influencing its chances for survival, rendering the two strains, although highly similar, in different physiological states that make one of them more fit for survival in host cells and consequently more pathogenic.

On the other hand, 10 membrane-associated proteins were observed with >5x or higher relative abundance in *M. tuberculosis *H37Ra. Only three of those (Rv0014c, Rv0070 and Rv1030), were proposed to have a biological function, the role of the rest is yet to be determined. The gene encoding transmembrane serine/threonine-protein kinase *pknB *(Rv0014c) protein was found to be essential for mycobacterial growth. This protein is thought to be involved in signal transduction via phosphorylation. *PknB *has been shown to be a substrate for phosphoserine/threonine phosphatase *PstP *(Rv0018c), which is also up-regulated in *M. tuberculosis *H37Ra, and its kinase activity is affected by *PstP*-mediated dephosphorylation. *PknB *and phosphoserine/threonine phosphatase *PstP *(Rv0018c) may act as a functional pair in vivo to control mycobacterial cell growth [50,51].

The putative gene *GlyA2 *(Rv0070) has been proposed to encode for the enzyme serine hydroxymethyltransferase (SHMT), up-regulated in *M. tuberculosis *H37Ra, is a pyridoxyl 5- phosphate (PLP)-dependent enzyme. The SHMT reaction plays a major role in cell physiology as it is considered to be a key enzyme in the pathway for interconversion of folate coenzymes that provide almost exclusively one-carbon fragments for the biosynthesis of a variety of end products such as DNA, RNA, ubiquinone, methionine, etc. [52]. The physiological role of SHMT is the reversible interconversion of serine to glycine. From the genome analysis of *M. tuberculosis*, there is an additional SHMT gene (GlyA1, Rv1093); the relative abundance of this enzyme is similar in both strains.

Moreover, the possible potassium-transporting p-type ATPase b (Rv1030) is also over 5 fold more abundant in *M. tuberculosis *H37Ra. This is one of the components of the high-affinity ATP-driven potassium transport system that catalyzes the hydrolysis of ATP coupled with the exchange of hydrogen and potassium ions. The gene encoding this protein was found to be non-essential for mycobacterial growth [53]. Taken together, these proteins and the ones with no defined physiological role present in higher amounts on the surface of *M. tuberculosis *H37Ra, provide a lead to elucidate the biological functions that might take us a step closer to understand the fundamental differences between the two strains and hence the mechanisms that influence pathogenicity.

**Figure 3:**
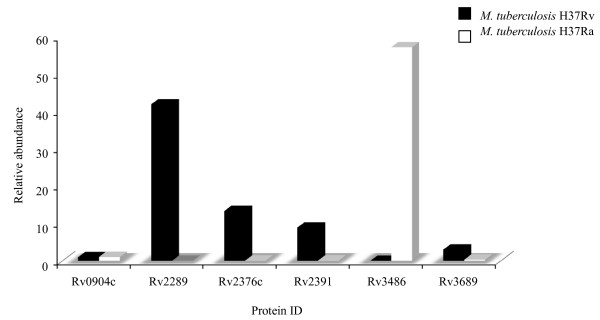
**Proteins reported by Gao et. al., (2004) to be consistently expressed at higher levels in H37Rv than in H37Ra, and are also observed in our study**.

Gao and colleagues (2004) [34], investigated the aggregation of mycobacteria into structures known as cords which is an intrinsic property of the human tubercle bacillus. This property is thought to be determined by the lipid composition of the bacterial cell surface and may contribute to the virulence of the organism [54]. Using microarray technology, they compared the pattern of gene expression of *M. tuberculosis *H37Rv with *M. tuberculosis *H37Ra under five different nutrient combinations and growth conditions. Under all of the conditions tested, *M. tuberculosis *H37Rv formed cords and *M. tuberculosis *H37Ra did not. By focusing their analysis only on genes that were differentially expressed under all conditions tested, they identified 22 genes that were consistently expressed at higher levels in H37Rv than in H37Ra. In our study we have observed 5 of those proteins, where 4 of them were observed in both strains, and one only in *M. tuberculosis *H37Rv strain. Interestingly, 5 proteins had a relative abundance higher than 5 fold in *M. tuberculosis *H37Rv which is in line with Gao's report, however, one of them (Rv2289) were >5x more abundant in *M. tuberculosis *H37Ra (Figure 3). This indicates that RNA level for genes are not directly proportional with the protein level, emphasizing the importance of transcriptome validation at protein level [55,56].

**Figure 4:**
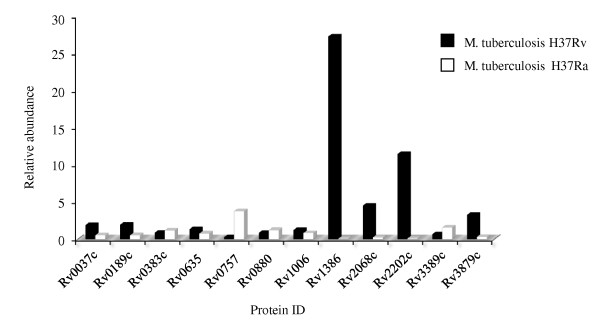
**Illustration showing proteins identified in this study reported by Zheng et. al., (2008)**.

In a comparative genome analysis of *M. tuberculosis *H37Rv and H37Ra to determine the basis of attenuation of virulence in H37Ra, Zheng and colleagues (2008) reported 57 genetic sequence variations between the two strains. They suggested that these variations may account for the attenuation of virulence in *M. tuberculosis *H37Ra and various other phenotypic changes that are different from its virulent counterpart *M. tuberculosis *H37Rv. Interestingly, the majority of these variations occurred in proteins thought to be exported to the membrane or involved in cell wall metabolism. We observed 12 of them, of which were up-regulated in *M. tuberculosis *H37Rv, while 7 had similar expression. Contrary to the expectation, we observed a 3.7 fold higher relative abundance in *M. tuberculosis *H37Ra (Figure 4) for the two-component transcriptional response regulator *PhoP *(Rv0757), which is reported to be associated with pathogenesis of *M. tuberculosis *H37Rv [57-59]. Frigui *et al.*, (2008) reported that a point mutation (S219L) in the predicted DNA binding region of the regulator *PhoP *is involved in the attenuation of H37Ra via a mechanism that influence the secretion of the major T cell antigen ESAT-6 [58]. *PhoP *controls the expression of many genes involved in the biosynthesis of complex cell wall lipids [59]. These proteins showed a less than 5-fold difference in our data. This observation is in line with the recent findings reported by de Souza et. al. (2010) [11], where they used label-free proteomic method to identify differentially abundant proteins in two closely related hypo- and hyper-virulent clinical *M. tuberculosis *Beijing isolates.

## Conclusion

Through a label-free proteomic analysis of the lipophilic proteins of the virulent *M. tuberculosis *H37Rv and its attenuated counterpart *M. tuberculosis *H37Ra, we showed that the two strains are highly similar at protein level. Our data confirm some of the findings that have been reported at the genomic level and we also show that the *PhoP *transcription factor is similar in both strains. In addition, our data suggest a role for secretion system subunit *SecF*, and ABC-transporter proteins as major differences between the two strains. To conclude, in light of what has been previously reported, this study extends the list of the potential determinants of differences in virulence between the two strains and adds to the current understanding of *M. tubeculosis *pathogenesis.

## Authors' contributions

HM performed protein extraction, data analysis and drafted the manuscript. GS carried out the search and quality control of the mass spectrometry analysis. SP cultured and harvested bacilli. TS performed protein digestion and preparation for mass spectrometry analysis. HW participated in result analysis, drafting the manuscript and overall design of the study. All authors read and approved the final manuscript.

## Supplementary Material

Additional file 1 **MTB H37Rv**. List of all *M. tuberculosis *H37Rv proteins identified in this study including their relative intensity. Click here for file

Additional file 2 **MTB H37Ra**. List of all *M. tuberculosis *H37Ra proteins identified in this study including their relative intensity. Click here for file

Additional file 3 **Membrane proteins**. List of all membrane proteins identified in one or both strains including their relative intensity and ratio. Click here for file

Additional file 4 **Lipoproteins**. List of all lipoproteins identified in one or both strains including their relative intensity and ratio. Click here for file

Additional file 5 **Differentially observed proteins**. List of all identified with a differential relative abundance of five times or more in one strain or the other. Click here for file

Additional file 6 **Additional Figure 1**. Collision induced disassociation fragmentation pattern of ion M+2H 1210.62. The sequence identified by the Mascot engine was LVLGSADGAVYTLAK from protein Rv2138. Click here for file
