# Supplementary figures and images for: Comparison of membrane proteins of Mycobacterium tuberculosis H37Rv and H37Ra strains
Source: BMC Microbiol. 2011 Jan 24;11:18. doi: 10.1186/1471-2180-11-18 (PMC3033788; doi:10.1186/1471-2180-11-18)

## Slide 1
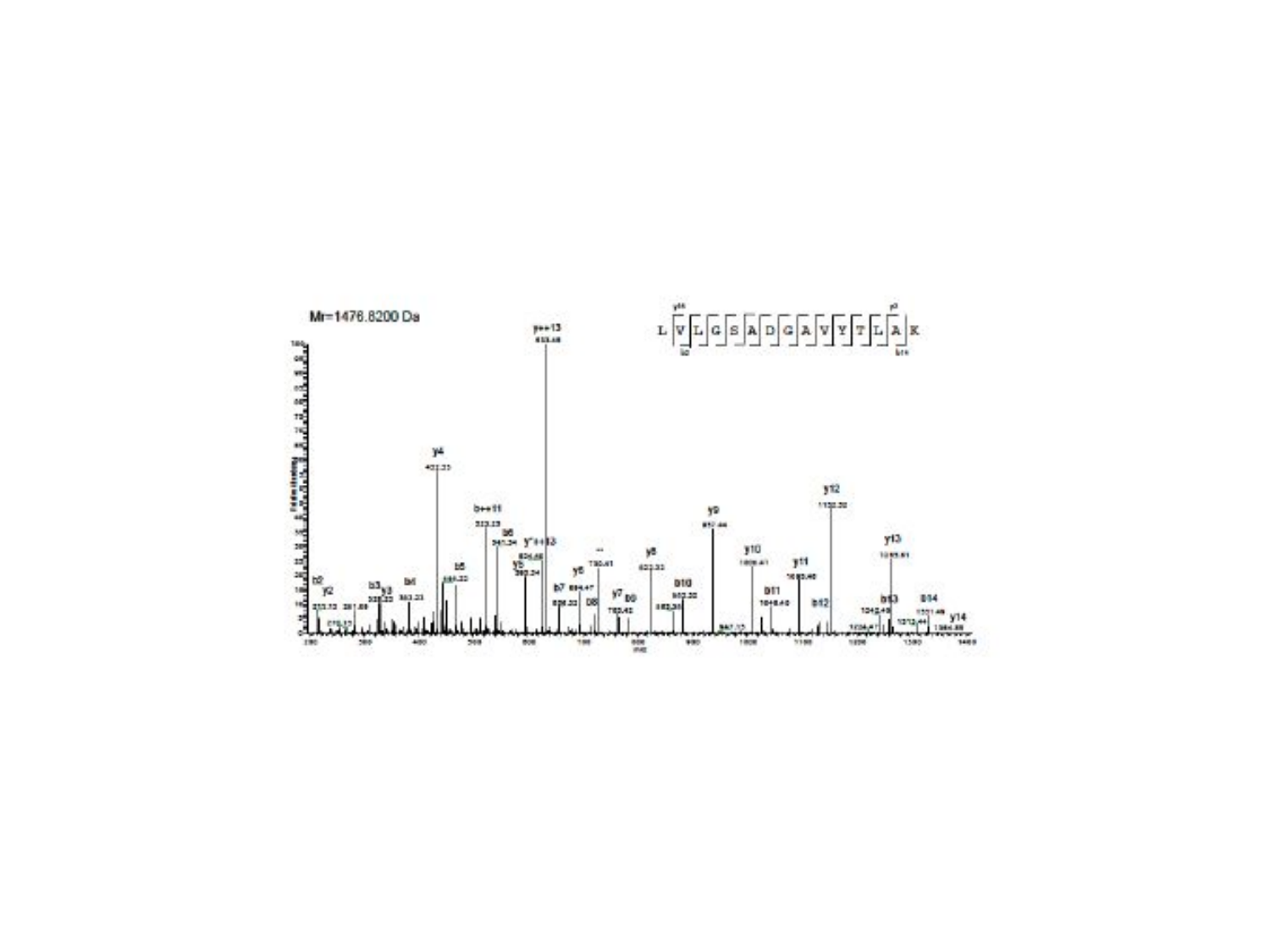

Supplement: Additional file 6 — Additional Figure 1. Collision induced disassociation fragmentation pattern of ion M+2H 1210.62. The sequence identified by the Mascot engine was LVLGSADGAVYTLAK from protein Rv2138. [file 1471-2180-11-18-S6.PPT]
